# Supplementary material for: SARS-CoV-2 RNA and antibody dynamics in a Dutch household study with dense sampling frame
Source: Sci Rep. 2022 May 13;12:7937. doi: 10.1038/s41598-022-11480-6 (PMC9099349; doi:10.1038/s41598-022-11480-6)
Supplement: Supplementary file 1 — Supplementary Information. [file 41598_2022_11480_MOESM1_ESM.pdf]

Table S1

Positivity and severity by age class			
	All	Adult	Children
cohort			
n	242	125	117
age	27 (SD=18)	42 (SD=11)	10 (SD=4)
gender (male)	114 (47%)	59 (47%)	55 (47%)
Visit 1			
Asymptomatic	66 (27%)	17 (14%)	49 (42%)
Mild	119 (49%)	60 (48%)	59 (50%)
Moderate	40 (17%)	34 (27%)	6 (5%)
Severe	11 (5%)	11 (9%)	0 (0%)
PCR NP pos	46 (25%)	25 (32%)	21 (20%)
PCR OP pos	45 (24%)	24 (31%)	21 (19%)
PCR Feces pos	71 (31%)	48 (39%)	23 (21%)
PCR oral fluid pos	59 (27%)	41 (36%)	18 (17%)
Microarray S1 pos	29 (13%)	25 (20%)	4 (4%)
Microarray N pos	34 (15%)	32 (26%)	2 (2%)
Wantai pos	79 (36%)	61 (50%)	18 (18%)
Visit 2			
Asymptomatic	97 (40%)	32 (26%)	65 (56%)
Mild	97 (40%)	59 (47%)	38 (32%)
Moderate	24 (10%)	22 (18%)	2 (2%)
PCR NP pos	28 (13%)	22 (18%)	6 (6%)
PCR OP pos	18 (8%)	11 (9%)	7 (7%)
PCR Feces pos	35 (15%)	15 (13%)	20 (18%)
PCR oral fluid pos	8 (4%)	6 (6%)	2 (2%)
Microarray S1 pos	83 (38%)	62 (50%)	21 (22%)
Microarray N pos	80 (36%)	65 (52%)	15 (15%)
Wantai pos	113 (54%)	84 (69%)	29 (33%)
Visit 3			
Asymptomatic	163 (67%)	74 (59%)	89 (76%)
Mild	27 (11%)	20 (16%)	7 (6%)
Moderate	9 (4%)	9 (7%)	0 (0%)
Severe	4 (2%)	3 (2%)	1 (1%)
PCR NP pos	0 (NaN%)	0 (NaN%)	0 (NaN%)
PCR OP pos	0 (NaN%)	0 (NaN%)	0 (NaN%)
PCR Feces pos	6 (3%)	3 (3%)	3 (3%)
PCR oral fluid pos	2 (1%)	1 (1%)	1 (1%)
Microarray S1 pos	90 (43%)	68 (57%)	22 (25%)
Microarray N pos	86 (41%)	66 (55%)	20 (22%)
Wantai pos	88 (59%)	75 (69%)	13 (32%)

Table S1. Study characteristics. Description age (mean; (sd)), gender (n male; (%)), symptom severity (n; (%)) and SARS-CoV-2 positive diagnosis per test (n; (%)) for the different age cohorts.

Figure S1

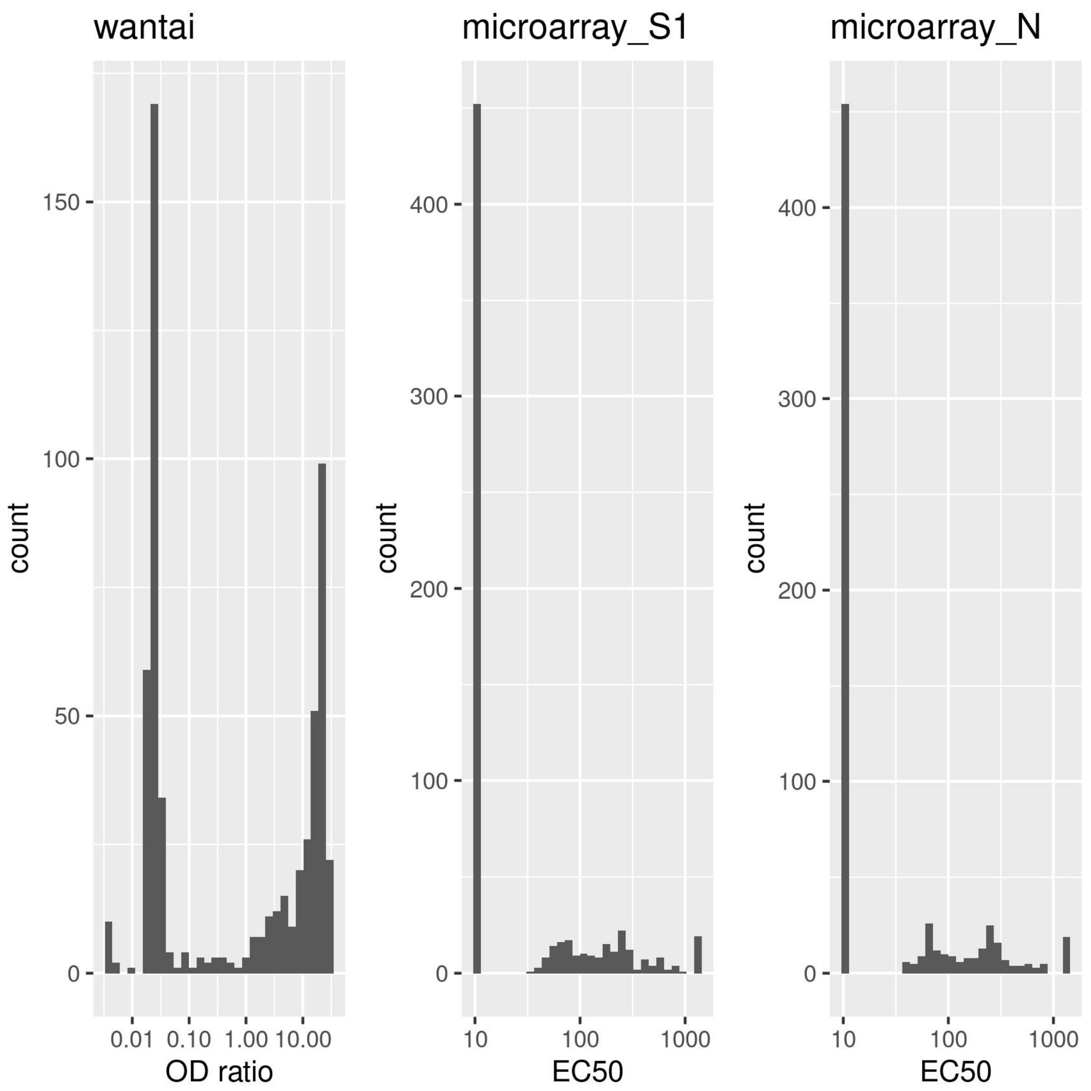

Figure S1. Histogram of Wantai OD ratios and microarray EC50 values. A clear separation between negative and positive components can be observed, with Wantai OD ratio ≥ 1.00 = positive and microarray EC50 > 10 = positive.

Figure S2

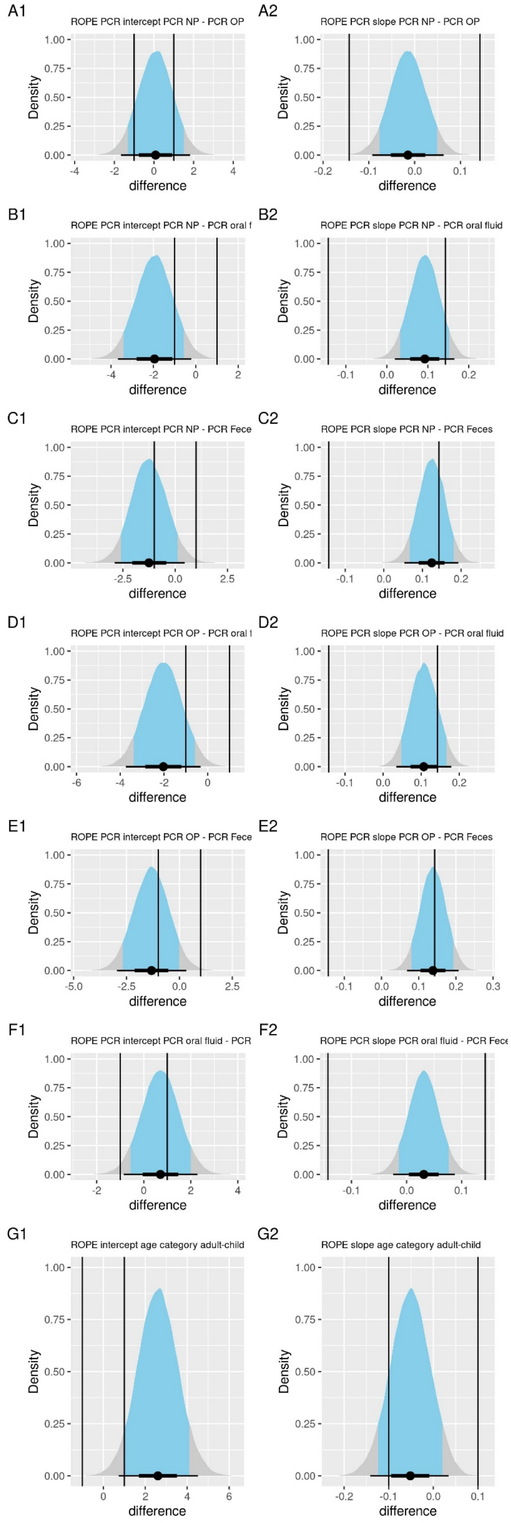

Figure S2: ROPE analyses for assessing differences between factors in Figure 5. For Ct-values our ROPE interval is [-1,1] (vertical lines, left panels A1 – G2), which means that we consider differences between Ct-values of less than one as not meaningful. For changes in Ct-value per day (the slope) we choose an interval of [-1/7, 1/7] (vertical line, right panels A2 – G2), which means that we consider differences between Ct-values of less than one per week as not meaningful. When the ROPE is outside the 89% highest posterior density interval (HDI) (blue area), there is a difference.

Figure S3

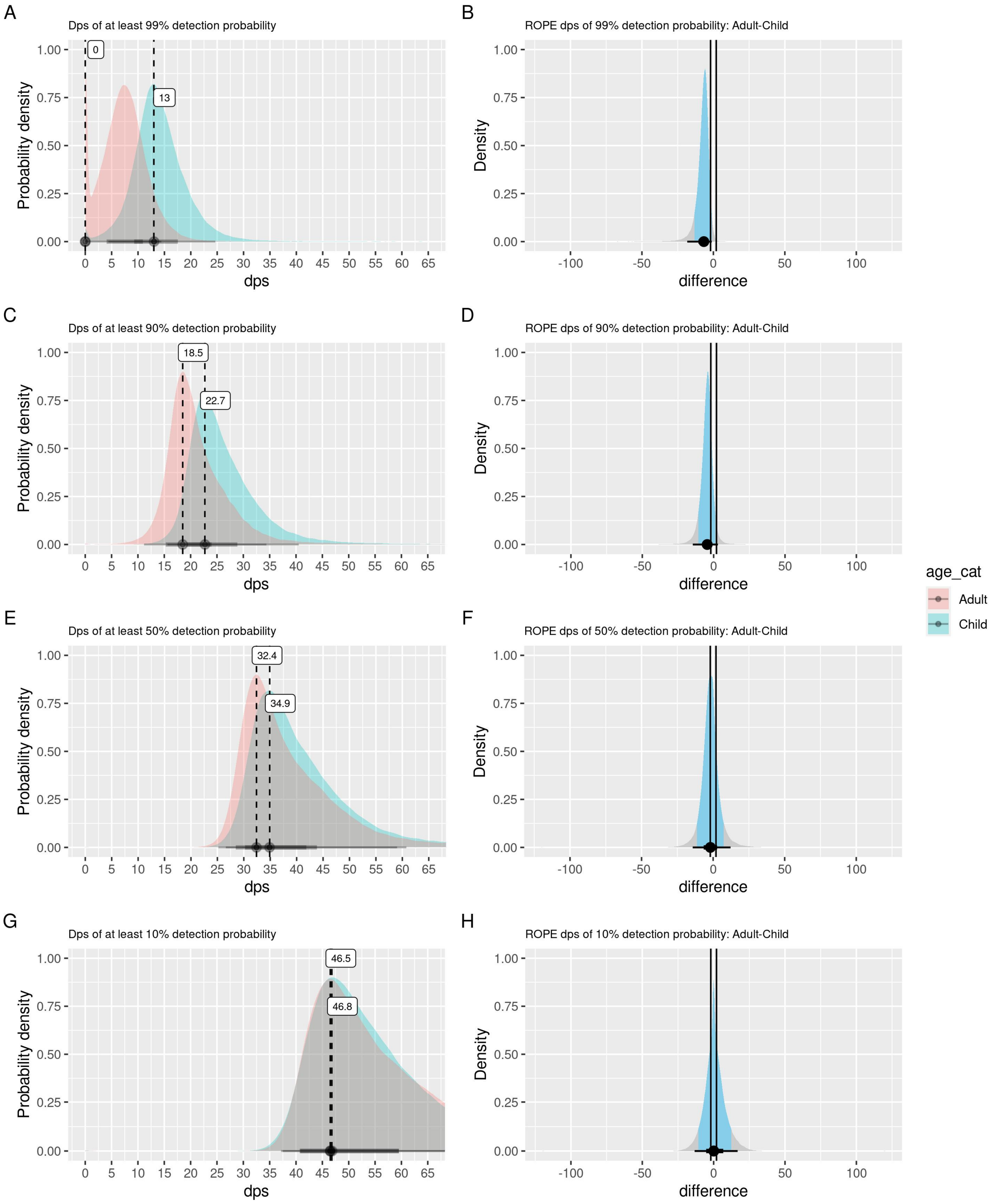

Figure S3: Dynamics SARS-CoV-2 infection diagnosis by RT-PCR in adults and children. Left panels: Average dps until when adults and children have detection probability of at least 99% (A), 90% (C), 50% (E) or 10% (G). Right panels: ROPE analyses for assessing differences between adults and children. Analyses for dps of 99% (B), 90% (D), 50% (F) and 10% (H) detection probability are shown. For Ct-values our ROPE interval is [-1,1] (vertical lines), which means that we consider differences between Ct-values of less than one as not meaningful. When the ROPE is outside the 89% highest posterior density interval (HDI) (blue area), there is a difference.

Figure S4

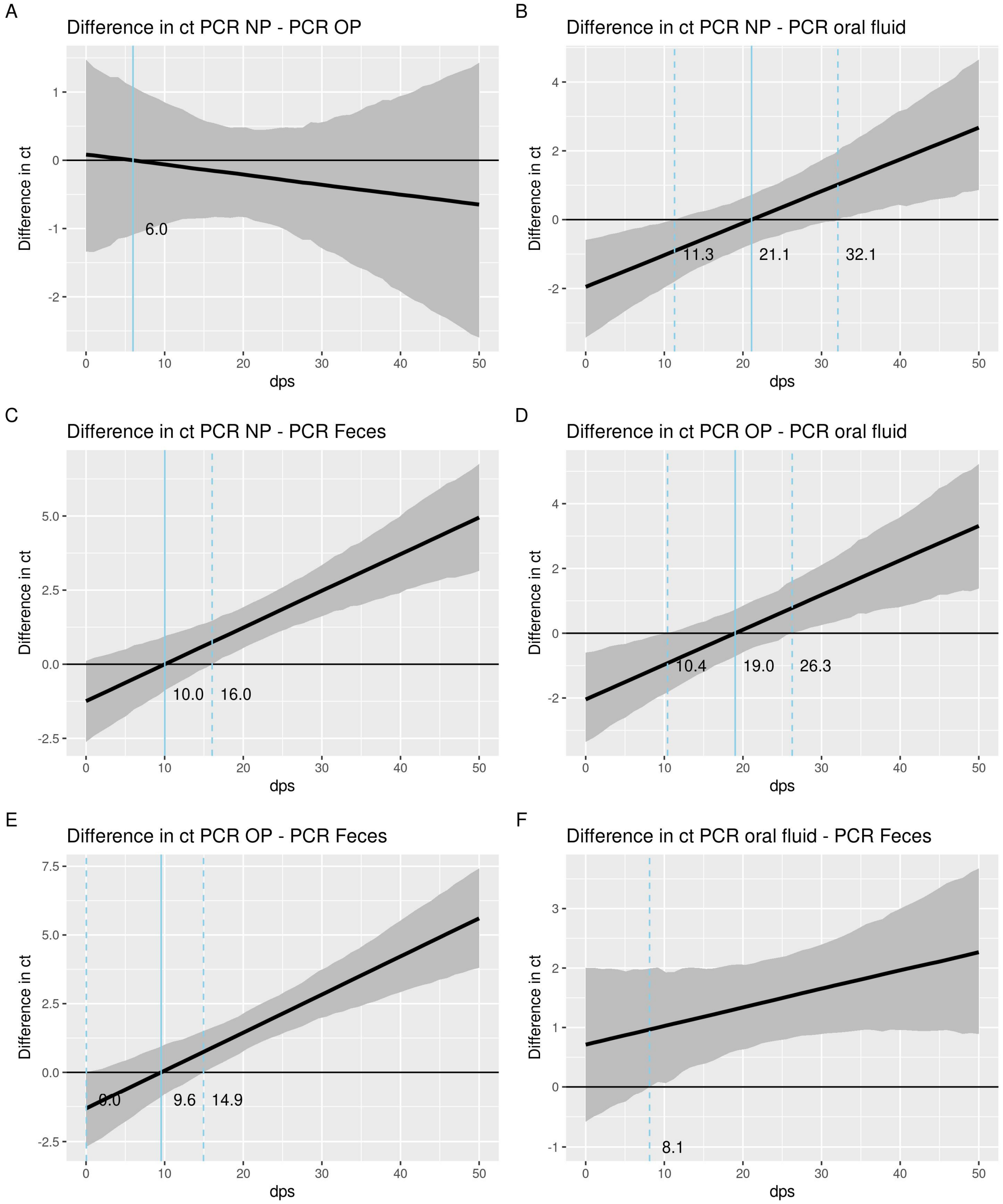

Figure S4: Differences in Ct between RT-PCR specimens in relation to dps. The black solid line indicates the median difference between the two assays (assay A – assay B) indicated, the shaded region is the 89% Bayesian credible interval. For reference the line y=0 is drawn, where this line crosses the solid line of median difference, the Ct values of the two assays are equal. The dps corresponding to this crossing point is indicated by a blue solid vertical line. The dashed lines indicate where y=0 crosses the top and bottom quantiles of the credible interval. To the left of the leftmost dashed line, we are sure that assay A has a smaller Ct then assay B, and conversely larger to the right of the rightmost line. In between those lines we are not certain, although more area of the credible interval below zero gives more credibility to a smaller Ct for assay A.

Figure S5

ROPE dps of 90,50,10% detection probability.

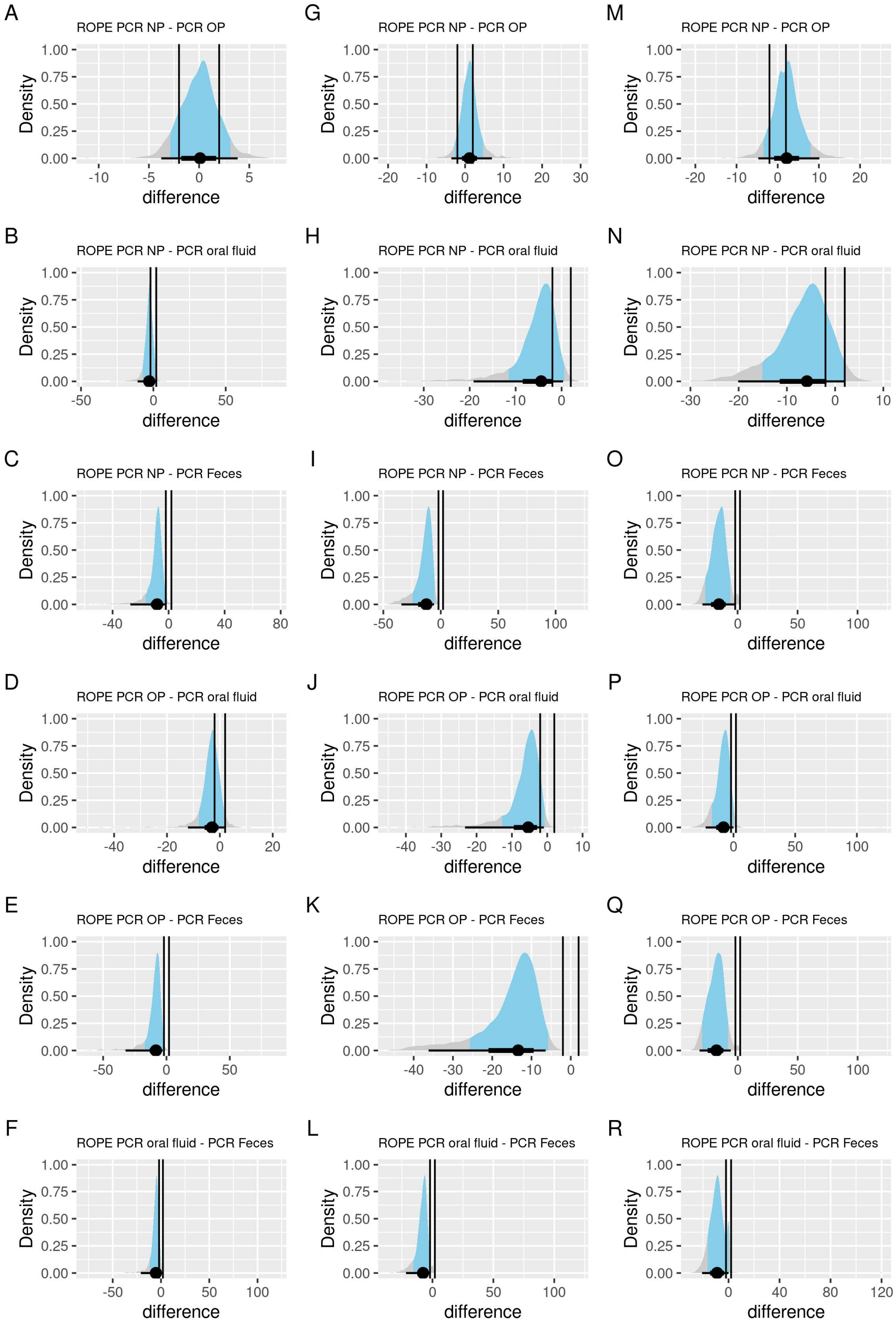

Figure S5: ROPE analyses for assessing differences between factors in Figure 5D and S4. Analyses for dps of 90% detection probability (left panels, A-F), 50% probability (middle panels, G-L) and 10% probability (right panels, M-R) are shown. For Ct-values our ROPE interval is [-1,1] (vertical lines), which means that we consider differences between Ct-values of less than one as not meaningful. When the ROPE is outside the 89% highest posterior density interval (HDI) (blue area), there is a difference.

Figure S6

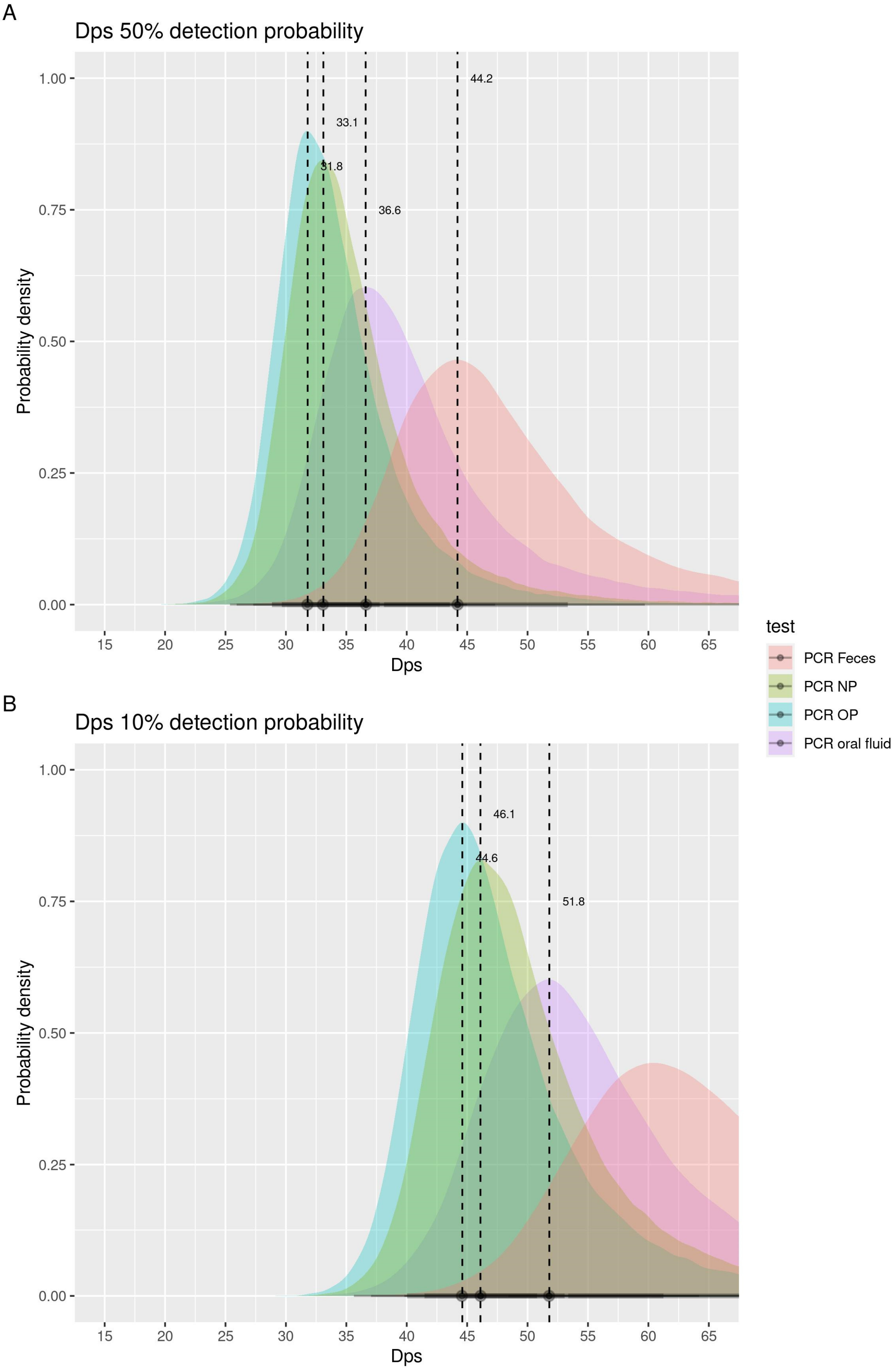

Figure S6: Dynamics SARS-CoV-2 infection diagnosis by RT-PCR in various specimens. Average dps until when different specimen have at least 50% (A) or 10% (B) detection probability.

Figure S7

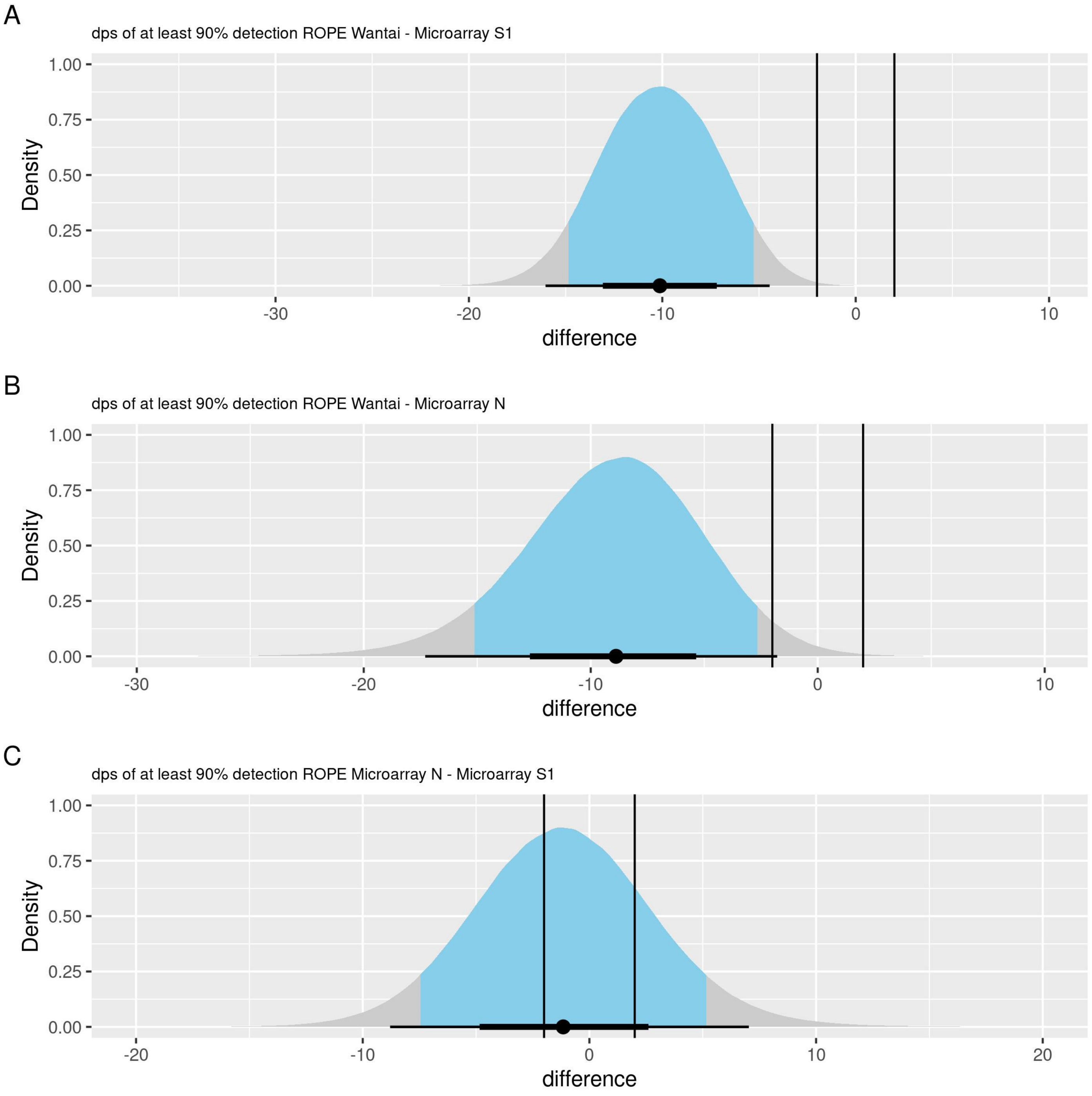

Figure S7: ROPE analyses for assessing differences between factors in Figure 6A and B. For serology detection probability (in dps) our rope interval is [-2,2], which means that we consider differences between days of less than 2 as not meaningful.

Figure S8

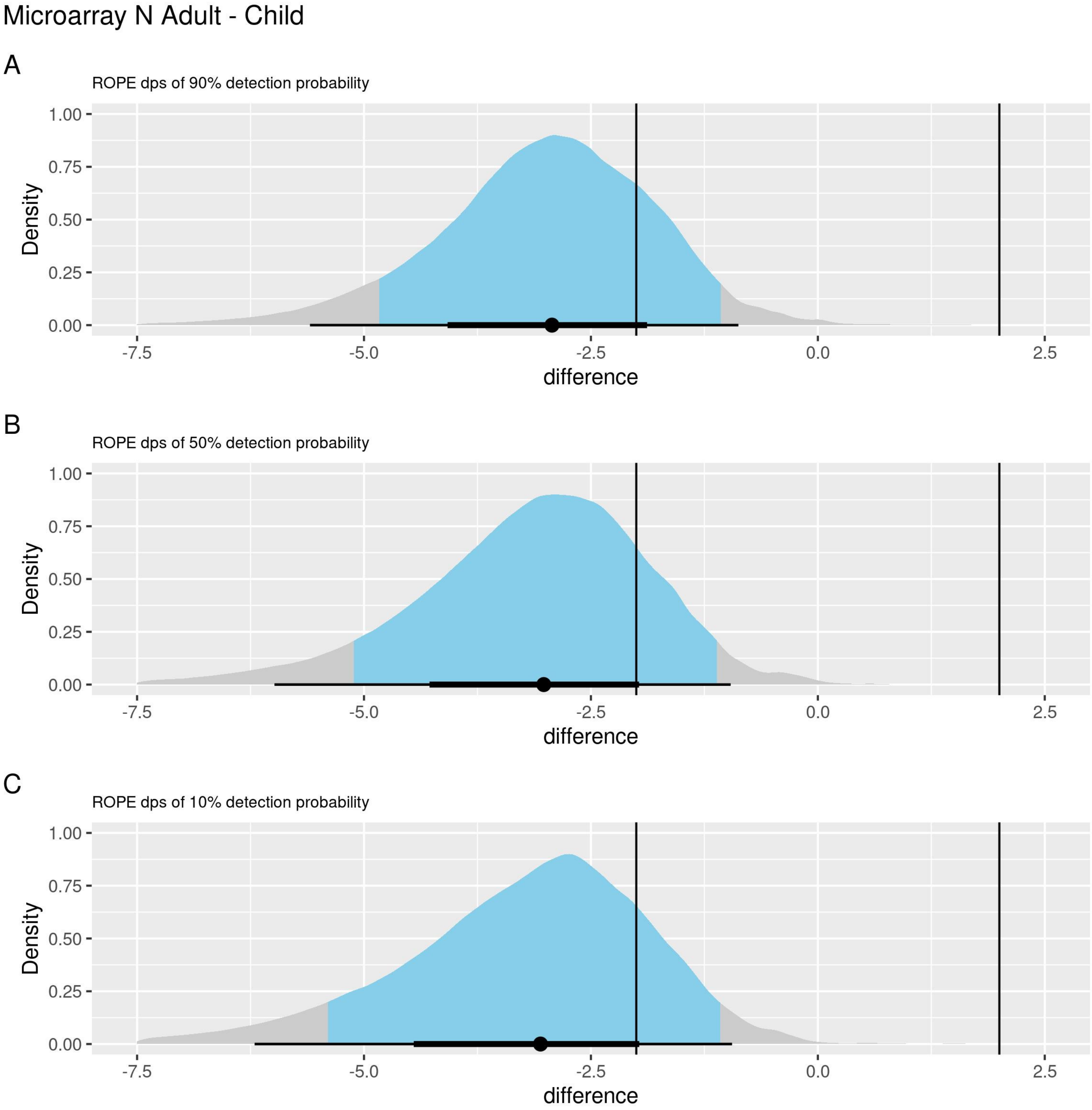

Figure S8: ROPE analyses for assessing differences between factors in Figure 6C and D. For serology detection probability (in dps) our rope interval is [-2,2], which means that we consider differences between days of less than 2 as not meaningful.

Figure S9

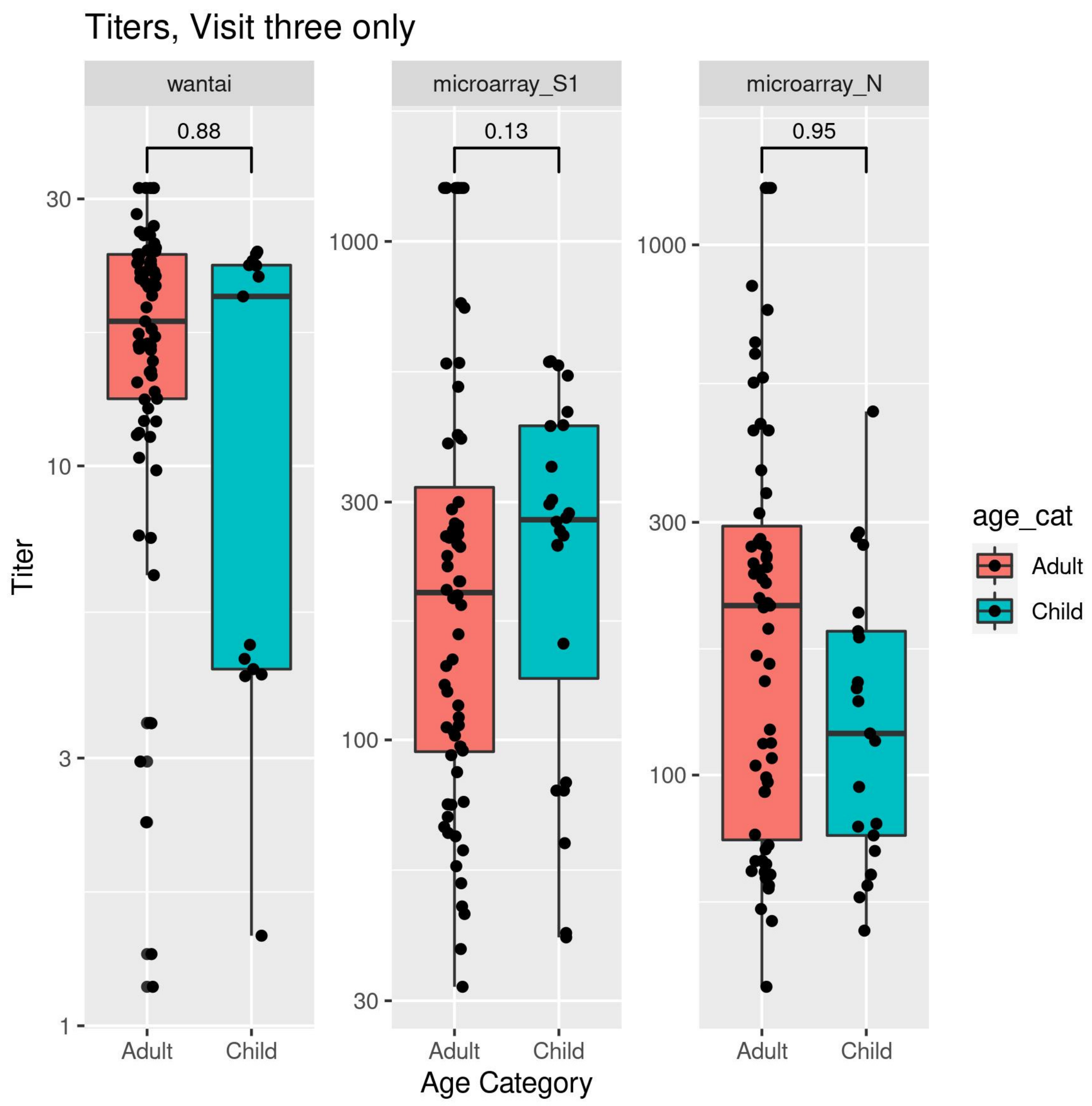

Figure S9: Effect of age on the development/magnitude of SARS-CoV-2 specific antibody responses. SARS-CoV-2 specific antibody titers at visit 3 (convalescence phase) in SARS-CoV-2 laboratory confirmed participants. SARS-CoV-2 Spike-specific IgM and IgG (Wantai, left), Spike-specific IgG (microarray S1, middle) and Nucleoprotein-specific IgG (microarray N, right) responses in relation to age (adult and child). On the Y-axis, titers in OD ratio (Wantai) and EC50 values (microarrays) are indicated. The boxplot compactly displays the distribution of a continuous variable. It visualizes five summary statistics (the median, two hinges and two whiskers). The lower and upper hinges correspond to the first and third quartiles (the 25th and 75th percentiles). The upper whisker extends from the hinge to the largest value no further than 1.5 * IQR from the hinge (where IQR is the inter-quartile range, or distance between the first and third quartiles). The lower whisker extends from the hinge to the smallest value at most 1.5 * IQR of the hinge. Differences were analyzed with the Wilcoxon test.

Figure S10

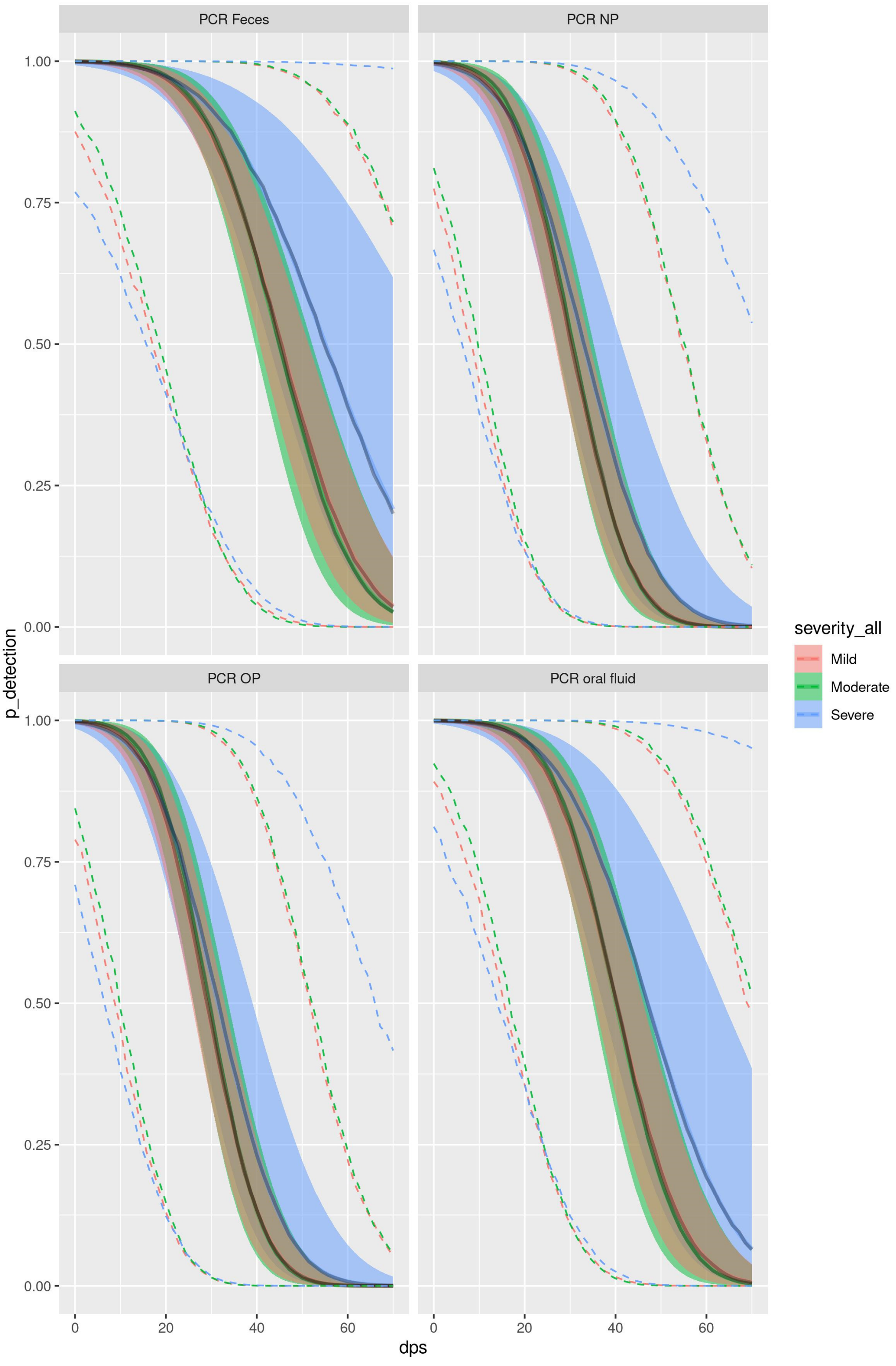

Figure S10: For each of the RT-PCR specimens, these graphs show the probability of detection as a function of days post symptoms. Shaded regions indicate Bayesian credible intervals, dotted lines indicate the 95% prediction interval. Different colors indicate the severity classes. No appreciable difference between the severity classes can be discerned, at reasonable dps values.

Figure S11

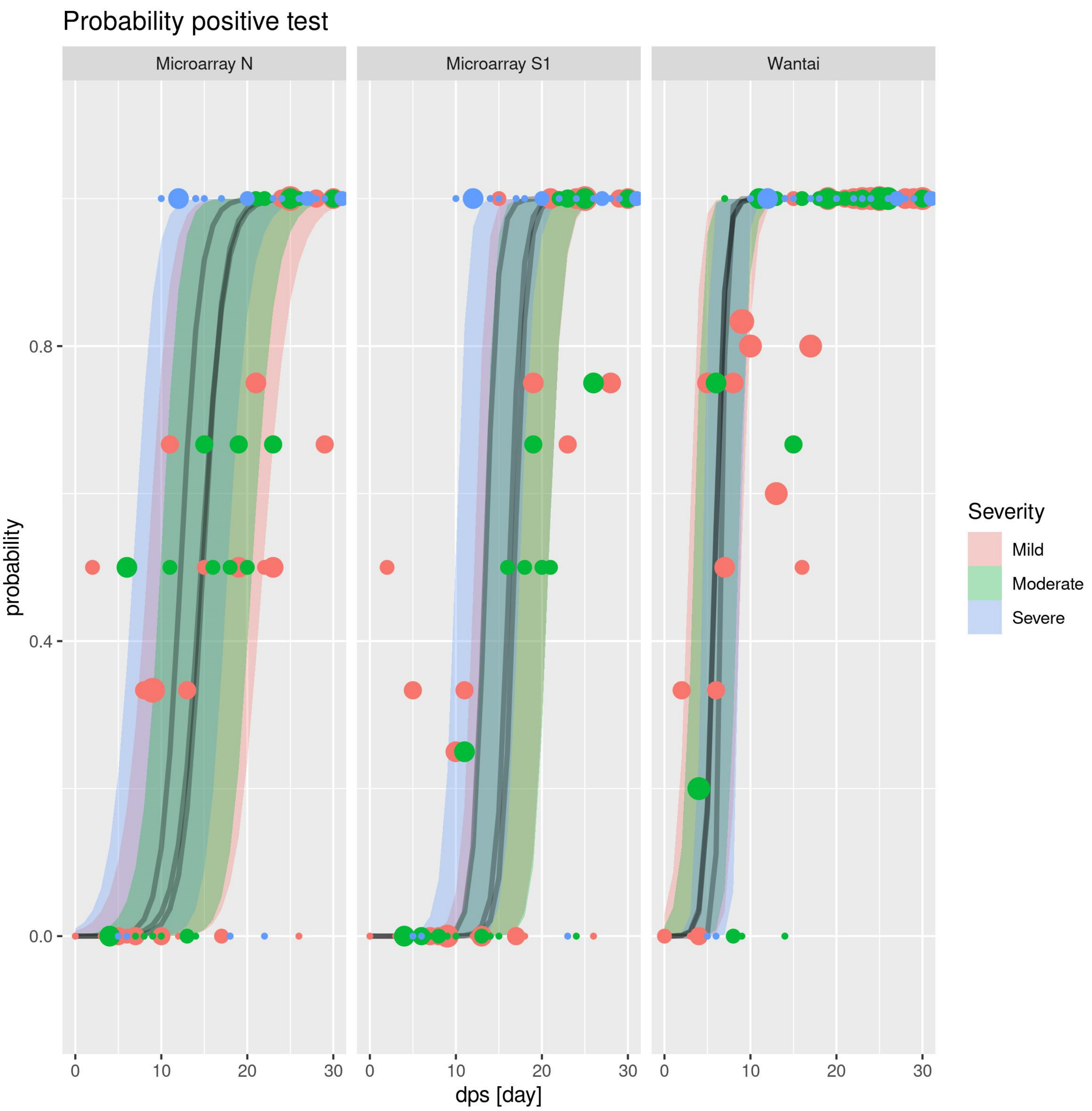

Figure S11: For each of serological assays, these graphs show the probability of detection as a function of days post symptoms. Shaded regions indicate Bayesian credible intervals. Different colors indicate the severity classes. No appreciable difference between the severity classes can be discerned.
